# Determinants of frailty among hospitalized older adults across various wards in a tertiary care hospital in Nepal

**DOI:** 10.1007/s40520-024-02895-4

**Published:** 2025-01-28

**Authors:** Prabha Shrestha, Lee Smith, Sarina Shakya, Yunika Acharya

**Affiliations:** 1 Department of Nursing, Kathmandu University School of Medical Sciences, Dhulikhel, Nepal; 2 Department of Public Health and Community Programs, Kathmandu University School of Medical Sciences, Dhulikhel, Nepal; 3 Global Brain Health Institute, University of California, San Francisco, USA; 4 Center for Health Performance and Wellbeing, Anglia Ruskin University, Cambridge, UK

**Keywords:** Frailty, Geriatric frailty, Hospitalized older adults, Tertiary hospital

## Abstract

**Introduction:**

Frailty, characterized by decreased resilience due to physiological decline, affects approximately 65% of community-dwelling elderly in Nepal. This study assessed frailty and its factors among hospitalized older adults in a tertiary hospital in Nepal.

**Methods:**

This cross-sectional study included 124 participants aged 60 and above, admitted to a tertiary hospital in Nepal. Frailty was assessed using the Groningen Frailty Index (GFI), a validated self-reported tool. Univariable and multivariable logistic regression analyses were conducted to identify factors associated with frailty, using STATA version 13.0.

**Results:**

Frailty was observed in 58.8% of participants. Adjusted analysis showed that, compared to those who cannot read and write, those with no formal education had 0.14 times lower odds of frailty (*p* = 0.01, 95% CI 0.03–0.66), while those with formal education had 0.19 times lower odds (*p* = 0.01, 95% CI = 0.04–0.73). Participants with comorbidities had 3.51 times higher odds of frailty (*p* = 0.01, 95% CI: 1.22–10.07), and those with a history of falls had 8.10 times higher odds (*p* = 0.005, 95% CI: 1.89–34.78).

**Conclusion:**

Frailty was prevalent in over half of the respondents. Lower levels of educational achievement, comorbidities, and a history of falls were identified factors of frailty. Targeted interventions addressing multimorbidity and fall prevention may reduce frailty risk among high-risk older adults in Nepal.

## Introduction

The global population is rapidly aging, reflecting the success of improved healthcare systems and public health interventions [1]. In Nepal, individuals aged 60 years and above are considered older adults, and the growth rate of this population surpasses that of the overall population [2]. In 2024, the life expectancy in Nepal reached 71.97 years, a 0.32% increase from 2023 [3]. According to the latest Census, older adults make up 10.21% of the total population [2]. With increasing life expectancy, additional years will likely be marked by a decline in both physical and mental abilities, extending beyond disease-related causes to include impairments in cognition, mood, and physical performance [4, 5]. This often leads to disability, dependence, and frailty.

Frailty is a clinical condition in which older adults face reduced resilience to everyday or acute stress due to age-related declines in physiological reserve and function across multiple organ systems [6]. Frailty is associated with a higher risk of falls [7], delirium [8], institutionalization [9], incident disability [10] and mortality [11]. Identification of older adults who are frail or at risk of being frail is of utmost importance. In Nepal, approximately 65% of the older community-dwelling participants self-reported the presence of frailty in the absence of disability [12]. A comparative study in Nepal reported that frailty was more prevalent among older people in old age homes (71.5%) compared to those in the community (56.3%) [13]. The living environment and lifestyle are key modifiable risk factors of frailty, both in old age homes and the community [13]. However, to the authors’ knowledge, no studies have been conducted to assess frailty among hospitalized older adults in Nepal.

Frailty is an increasingly prevalent geriatric syndrome that increases the risk of falls, delirium, dependency, and elevated mortality rates among older adults, while also increasing the risk of vulnerability to abuse and mistreatment [14]. Although frailty is preventable, most research focuses on community-dwelling older adults, with limited exploration of this condition in hospital settings. Hospitalized older adults, especially those with acute illnesses, are more susceptible to frailty [15, 16], however, the available evidence in this context is insufficient. Whilst numerous studies have established significant associations between frailty and factors such as hospitalization, hypertension, diabetes, and falls in Nepal, there is a scarcity of research that has examined frailty among hospitalized older adults in this setting. As frailty is a universal problem among elderly populations, the present study conducted in Nepal can provide meaningful information for other Low Middle Income Countries (LMICs) with comparable settings. Understanding these determinants can support the development and implementation of tailored frailty interventions suited to resource-limited environments, aiding LMICs in prioritizing better outcomes for older adults.

Therefore, this study aims to assess frailty and its influencing factors among older adults admitted to a tertiary-level hospital in Nepal. Given the multifaceted nature of frailty, this study applies the Biopsychosocial Model to explore the complex interplay of biological and social factors contributing to frailty among hospitalized older adults [17]. Based on this model, biological factors such as age and sex, presence of comorbidities, and social factors, including religion, and financial sources and the history of falls are considered key indicators that reflect the intersection of both biological and social factors.

## Methods

### Study design and settings

We conducted a cross-sectional survey of patients over 60 years of age who were admitted to different wards: medical, surgical, orthopedics, gynecology, and Eye, Nose, and Throat (ENT) in Dhulikhel Hospital tertiary-level community hospital on the outskirts of Kathmandu, Nepal. 

### Participants

A total of 124 patients were selected conveniently from various wards of Dhulikhel Hospital. Those patients with terminal illness, identified as “seriously ill” for more than 72 hours from the day of admission, and lacking mental capacity were excluded from the study. The sample size was calculated with G power software for binary logistic regression with an odd ratio of 1.7 obtained from a similar study conducted on Korean older adults [18]. 

Written consent was obtained from the participants. This study was approved by the Kathmandu University Institutional Review Committee (KU-IRC), approval number 57/22.

### Data collection

Trained research assistants conducted face-to-face interviews with the participants in Nepali from September 2022 to November 2023, using a structured questionnaire.

### Measures

Socio-demographic questions were adopted from a previously conducted national survey of Nepal [19]. Frailty was assessed by the Groningen Frailty Index (GFI). This validated instrument has been used to assess frailty and has been tested in multiple settings [20–22]. It has 15 items that assess four domains: physical (nine items), cognitive (one item), social (three items), and psychological (two items). This tool has been translated and applied to Nepalese older adults [13].

### Data analysis

Categorical data are reported as frequencies and percentages, and numerical data with means and standard deviations. We used univariable and multivariable logistic regression models to assess the association between socio-demographic and other variables with frailty. In the multivariable model, we adjusted the potential confounders based on a prior literature review. We reported crude and adjusted odds ratios with a 95% confidence interval and p-value. All analyses were conducted using STATA version 13.0 (Stata Corp., College Station, Texas, USA) for cleaning, coding, and statistical analysis.

## Result

Table 1 shows the socio-demographic characteristics of the participants. The mean age of participants was 72.6 (7.07) years. The percentage of males and females was nearly equal. The majority of the participants were Janjati ethnicity (44.3%) and Hindu religion (70%). Nearly 70% of the participants were married. The majority of the participants were not able to read and write (64.5%). More than 80% of the participants were not currently working. The major financial source of the participants was employment/post-employment income (76.6%). 72% of the participants had comorbidities. 20% of the participants had a history of falling in the past year.

**Table 1:** Socio- demographic characteristics of the participants (*n* = 124)

Characteristics	*n* (%)
Age Mean (SD)	72.6 (7.07)
**Sex**	
Male	63 (50.9)
Female	61 (49.1)
**Ethnicity**	
Brahmin/Chhetri	47 (37.9)
Janjati	55 (44.3)
Others	22 (17.8)
**Religion**	
Hindu	92 (74.1)
Non-Hindu	32 (25.9)
**Marital status**	
Married	86 (69.3)
Single	38 (30.7)
**Education level**	
Cannot read and write	80 (64.5)
No formal education	20 (16.1)
Formal education	24 (19.4)
**Financial source**	
Employment/post-employment income	95 (76.6)
Family	29 (23.4)
**Comorbidity**	
No	34 (27.5)
Yes	90 (72.5)
**History of fall in last year**	
No	97 (78.3)
Yes	27 (21.7)

N: Frequency %Percentage

Table 2 shows the frailty-related characteristics of the participants (*n* = 124). In mobility, more than half (69.3%) of the participants experienced difficulty with grocery shopping, 15% of the participants experienced difficulty with walking outside the house, 10.4% of the participants experienced problems in their daily life due to difficulty in walking and 12.1% of the participants experienced difficulty with getting undressed. Regarding their vision and hearing, 44% and 41% of the participants respectively experienced problems. Forty-four of the participants unintentionally lost weight in the past 6 months. 30% of the participants were using 4 or more different types of medication. 38% of the participants were complaining about his/her memory (or diagnosed with dementia). Regarding psychosocial, 35.5% of participants experience emptiness, 37.9% miss the presence of other people, 29.1% feel left alone, 36.2% feel down or depressed lately, and 27.5% feel nervous or anxious.

**Table 2:** Frailty-related characteristics of participants (*n* = 124)

Characteristics	*n* (%)	*n* (%)
	Yes	No
**Mobility**		
Grocery shopping	38 (30.7)	86 (69.3)
Walk outside the house (around the house or to the neighbor)	19 (15.3)	105 (84.7)
Experiencing problems in your daily life due to difficulty in walking	13 (10.4)	111(89.5)
Getting (un)dressed	15 (12.1)	109 (87.9)
**Vision**		
Encounter problems in daily life because of impaired vision	55 (44.3)	69 (55.6)
**Hearing**		
Encounter problems in daily life because of impaired hearing	51 (41.2)	73 (58.8)
**Nutrition**		
Unintentionally lost a lot of weight in the past 6 months (6 kg in 6 months or 3 kg in 3 months)?	55 (44.3)	69 (55.7)
**Comorbidity**		
Patients using 4 or more different types of medication	38 (30.7)	86 (69.3)
**Cognition**		
Patient complaining about his/her memory (or diagnosed with dementia)	48 (38.7)	76 (61.3)
**Psychosocial**		
Patients experiencing emptiness	44 (35.5)	80 (64.5)
Ever miss the presence of other people	47 (37.9)	77 (62.1)
Feeling down or depressed lately	45 (36.2)	79 (63.8)
Felt nervous or anxious lately (in the past month)	45 (36.2)	90 (72.5)
**Physical Fitness**	58 (47.1)	65 (52.8)

N: Frequency %Percentage

**Fig. 1:**
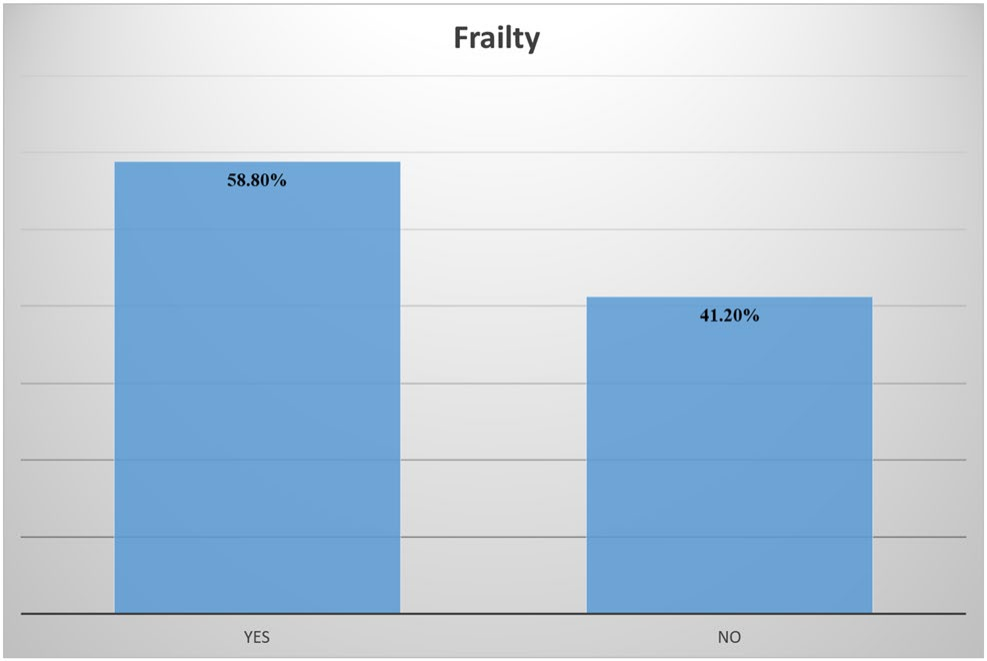
Presence of frailty

More than half of the participants (58.8%) have experienced frailty (Fig. 1).

Table 3 shows the factors associated with frailty among study participants. After adjusting for the confounders, as compared to participants who cannot read and write, the odds of experiencing frailty were 0.14 times lower among those participants who had no formal education (*p* = 0.01, 95% CI 0.03–0.66), and 0.19 times lower among those who had formal education (*p* = 0.04–0.73). The odds of experiencing comorbidities were 3.51 times higher among those with comorbidities compared to those without the presence of comorbidity (*p* = 0.01, 95% CI 1.22–10.07). The odds of experiencing frailty were 8.10 times higher among those who had a history of falls in the past year compared to those who did not (*p* = 0.005, 95% CI 1.89–34.78).

**Table 3:** Factors associated with frailty among study participants (*n* = 124)

Characteristics	Univariable			Multivariable		
	OR	*p*-value	95% CI	*aOR	*p*-value	95% CI
**Age**	1.04	0.13	0.98–1.09	1.01	0.65	0.94–1.09
**Sex**						
Male	Ref			Ref		
Female	3.51	0.001	1.64–7.49	0.79	0.71	0.24–2.63
**Ethnicity**						
Brahmin/Chhetri	Ref			Ref		
Janjati	1.67	0.20	0.75–3.70	1.62	0.32	0.62–4.26
Others	1.67	0.33	0.59–4.74	1.59	0.49	0.42–6.02
**Marital status**						
Single	Ref			Ref		
Married	0.39	0.02	0.16–0.90	0.75	0.61	0.24–2.29
**Educational status**						
Cannot read and write	Ref			Ref		
No formal education	0.25	0.008	0.09–0.70	0.14	**0.01**	0.03–0.66
Formal education	0.15	< 0.01	− 0.05-0.42	0.19	**0.01**	0.04–0.73
**Financial source**						
Employment and post-employment income	Ref			Ref		
Family income	0.68	0.37	0.29–1.57	0.80	0.69	0.26–2.42
**Comorbidity**						
No	Ref			Ref		
Yes	5.6	< 0.01	2.35–13.29	3.51	**0.01**	1.22–10.07
**History of fall in the past year**						
No	Ref			Ref		
Yes	7.83	0.001	2.21–27.75	8.10	**0.005**	1.89–34.78

Adjusting for age, sex, ethnicity, marital status, financial source, comorbidity, and history of fall in the last year

## Discussion

Among hospitalized patients, frailty was prevalent among more than half of the participants. Compared to those who cannot read and write, those who had no formal education and those with formal education were less likely to have frailty. Whereas, those with comorbidities and those who had a history of falls in the past year were more likely to have frailty.

In the present study, over half of the participants were found to have frailty, which is consistent with findings from a community-based study in Nepal (56.3%) [13]. However, the prevalence observed in our study is lower compared to that reported in a study involving individuals living in old age homes [13]. Interestingly, the prevalence in this study is higher as compared to a study conducted among pensioners residing in Nepal which found a prevalence of 46.2% [23]. The prevalence rate of frailty in China, and Indonesia is 18.02% and 25.2% respectively [18, 24]. These differences can be attributed to a variety of factors that participants are exposed to, such as social support, social interaction, mobility, and daily lifestyle [25–27]. The lower prevalence of frailty in the study including pensioners residing in Nepal may be owing to the benefit of socio-economic conditions and healthcare access [28]. These differences highlight the significant role that environmental, social, and health-related factors play in the prevalence of frailty.

In this study, a significant association between the lower level of educational achievement and frailty was observed, aligning with findings from previous research. This is consistent with a study conducted in Brazil, which demonstrated a similar relationship, where individuals with lower levels of educational achievement were more likely to experience frailty [29, 30]. Similar results were found in a longitudinal study conducted in Amsterdam, lower level of education achievement was found to be a determinant of frailty over time [30]. These associations may be explained by higher educational achievement being associated with better access to health information, healthier lifestyles, and improved social and economic conditions, which often contribute to maintaining physical and cognitive health in older age [31]. The consistency of our findings with studies from diverse geographic regions suggests that the link between education and frailty is robust across different populations and settings.

This study also found a significant association between frailty and the presence of comorbidities which is similar to the study conducted in Nepal among pensioners where various health issues were significantly associated with frailty [23]. Similarly, a study focusing on older adults in Nepal identified that health issues such as diabetes and hypertension were prevalent among frail individuals [32]. These findings suggest that the presence of chronic diseases may exacerbate frailty by reducing physical resilience and increasing vulnerability to adverse health outcomes.

The present study also found a significant association between frailty and a history of falls, a finding consistent with previous research conducted among older adults with type 2 diabetes mellitus [33]. Indeed, falls can lead to reduced mobility, loss of independence, and a decline in physical function, all of which contribute to frailty [34–36]. Furthermore, the fear of falling may limit physical activity, further accelerating the progression of frailty.

This study did not observe an association between age, sex, or marital status with frailty. Similarly, a longitudinal study conducted in Germany also did not find a significant association with marital status which is consistent with the present findings [37]. However, the study found an association with age. In the Kathmandu Valley, one study found that individuals over 80 years of age showed higher frailty scores [13]. This might be due to cultural, lifestyle, and healthcare access factors of the study setting that might mitigate the influence of age and marital status on frailty, compared to other populations where these variables have shown associations. Another reason might be due to variations in study design, sample size, and methods of frailty assessment leading to the differences in findings across studies.

### Strengths and limitations of the study

This study offers valuable insights into frailty among the geriatric population and its associated factors in hospitalized patients. The findings are useful for designing targeted programs to address and prevent frailty. Additionally, the study demonstrates an independent association between frailty and various exposures, even after adjusting for potential confounders. However, the study has some limitations. First, as a cross-sectional study, it does not allow for establishing causality. Thus, some of the observed associations may be bi-directional. Second, because the research was conducted at a single location, the results may not be generalizable to broader Nepal populations.

## Conclusion

This study found a substantial proportion of frailty suggesting a need for immediate action. Those with lower level of educational achievement, presence of comorbidities, and a history of falls in the last year were more likely to experience frailty. The findings suggest that further research is necessary to explore participant’s perspectives on their lived experiences with frailty and to design targeted health interventions. It is recommended that authorities prioritize managing multimorbidity and implementing fall prevention strategies to reduce frailty risk in this group. Additionally, post-discharge screening programs for managing hearing and vision impairments should be developed and implemented to lower the risk of frailty.

## Data Availability

Data are available upon reasonable request.
